# Evaluation of Association Studies and Meta-Analyses of *eNOS* Polymorphisms in Type 2 Diabetes Mellitus Risk

**DOI:** 10.3389/fgene.2022.887415

**Published:** 2022-06-27

**Authors:** Di Wang, Liangshu Liu, Chengyu Zhang, Wensheng Lu, Feifei Wu, Xiaofeng He

**Affiliations:** ^1^ Changzhi Medical College, Changzhi, China; ^2^ Department of Endocrinology and Metabolism, Guangxi Academy of Medical Sciences and the People’s Hospital of Guangxi Zhuang Autonomous Region, Nanning, China; ^3^ Department of Endocrinology and Metabolism, Heping Hospital Affiliated to Changzhi Medical College, Changzhi, China; ^4^ Department of Epidemiology, School of Public Health, Southern Medical University, Guangzhou, China; ^5^ Institute of Evidence-Based Medicine, Heping Hospital Affiliated to Changzhi Medical College, Changzhi, China

**Keywords:** eNOS, polymorphism, T2DM, meta-analysis, BFDP, FPRP

## Abstract

**Background:** Numerous studies reported the associations between endothelial nitric oxide synthase (*eNOS*) polymorphisms (4b/a VNTR (rs869109213), G894T (rs1799983) and T786C (rs2070744)) and type 2 diabetes mellitus (T2DM) risk. However, the conclusions were incongruent. Moreover, since no published meta-analyses were performed, a key issue regarding false-positive results needs to be addressed. Furthermore, four new articles have been published on these issues. Therefore, an updated meta-analysis was conducted to further explore these associations.

**Objectives:** To investigate the association between *eNOS* 4b/a, G894T and T786C polymorphisms and T2DM risk.

**Methods:** Studies were searched by using the PubMed, China National Knowledge Infrastructure (CNKI), Medline, Embase, International Statistical Institute (ISI) and the China Wanfang databases. Odds ratios (ORs) with 95% confidence intervals (CIs) were used to evaluate the associations using five genetic models. Furthermore, the false-positive report probability (FPRP), Bayesian false discovery probability (BFDP), and the Venice criteria were employed to assess the credibility of statistically significant associations.

**Results:** Overall, the *eNOS* 4b/a polymorphism was associated with a significantly decreased T2DM risk in Asians (bb vs. aa: OR = 0.44, 95% CI = 0.23–0.84; ab + bb vs. aa: OR = 0.45, 95% CI = 0.24–0.86; bb vs. aa + ab: OR = 0.73, 95% CI = 0.59–0.91; b vs. a: OR = 0.71, 95% CI = 0.57–0.88); the *eNOS* G894T polymorphism was associated with a significantly increased T2DM risk in Asians (GT vs. GG: OR = 1.52, 95% CI = 1.15–2.01; GT + TT vs. GG: OR = 1.52, 95% CI = 1.15–2.01; T vs. G: OR = 1.39, 95% CI = 1.09–1.76); the *eNOS* T786C polymorphism was associated with a significantly increased T2DM risk in Indian (TC vs. TT: OR = 1.93, 95% CI = 1.27–2.94; TC + CC vs. TT: OR = 2.06, 95%CI = 1.26–3.36; C vs. T: OR = 1.90, 95%CI = 1.17–3.08). However, when a sensitivity analysis was performed after excluding low quality and Hardy–Weinberg Disequilibrium (HWD) studies, no significant association was found for the *eNOS* G894T polymorphism. After credibility assessment, we identified “less-credible positive results” for the statistically significant associations in the current meta-analysis.

**Conclusion:** In conclusion, this article suggests that all substantial relationships between *eNOS* 4b/a, G894T, and T786C polymorphisms and T2DM risk are most likely due to false positive results rather than real connections or biological variables.

## Introduction

Type 2 diabetes mellitus (T2DM), which is defined by chronic hyperglycemia caused by insulin resistance as well as multiple related micro-vascular and macro-vascular complications, is one of the most common chronic illnesses at home and abroad. Over the last 3 decades, the global prevalence of diabetes mellitus has more than quadrupled, making it one of the most serious global health issues (Moore et al., 2009). At the same time, it is reported that the incidence of T2DM is increasing at an alarming rate. Diabetes is anticipated to impact 702 million people by 2045, which means one in every eleven people will be affected, and huge amounts of money will be required globally to cure diabetes and manage its complications (https://diabetesatlas.org/en/). However, the pathogenesis of T2DM remains unclear and may be related to diet and exercise, obesity, geography, genetic susceptibility, environment, etc. Furthermore, there is abundant evidence that genetic predisposition plays a significant role in the etiology of T2DM (Ferland-McCollough et al., 2010). It has been reported that there is an important genetic predisposition for T2DM (Papazafiropoulou et al., 2017). At the same time, over 100 T2DM risk loci have been identified to date, although the molecular pathways of risk genes are unclear (Gaulton, 2017). In conclusion, genetic factors play an essential impact in the occurrence and development of T2DM.

Nitric oxide (NO) is a ubiquitous vasoactive substance, whose main function is to protect vascular endothelial cells from damage (Larsen et al., 2012). Endothelial dysfunction due to reduce in NO levels is an important mechanism for the development of T2DM. One of the essential enzymes in the process of NO generation is endothelial nitric oxide synthase (*eNOS*), which is encoded by the *eNOS* gene on chromosome 7q35-7q36 (Jamwal and Sharma, 2018). It has been observed that *eNOS* malfunction can cause a nitric oxide production problem, which can contribute to the development of characteristic T2DM aberrant metabolic phenotypes such as reduced glucose tolerance and insulin resistance (Li et al., 2002; Tsutsui et al., 2006). Therefore, *eNOS* polymorphisms may biologically be an ideal genetic marker for T2DM in biology.

Many eNOS gene polymorphisms have been identified in recent years, of which 4b/a, G894T, and T786C are the most investigated polymorphisms in T2DM (Wang et al., 1997; Veldman et al., 2002), although their associations remain controversial and equivocal. Several relevant meta-analyses have been performed to evaluate the correlations of T2DM with *eNOS* gene polymorphisms (Jia et al., 2013; Zhang et al., 2017; Dong et al., 2018), with conflicting results. And previously published meta-analyses did not evaluate the quality of the literature, nor did they evaluate the positive results to identify multiple comparisons. As a result, an updated meta-analysis was conducted to further investigate the possible association between *eNOS* genetic variants (4b/a, G894T, and T786C) and T2DM risk. This analysis included more papers and credible findings than previous meta-analyses (Jia et al., 2013; Zhang et al., 2017; Dong et al., 2018).

## Materials and Methods

### Search Strategy

The current study was performed according to the guidelines of the Preferred Reporting Items for Systematic Review and Meta-Analysis (PRISMA) group (Moher et al., 2009). The literature was searched using PubMed, China National Knowledge Infrastructure (CNKI), Medline, Embase, ISI (International Statistical Institute) and the China Wanfang databases. The following search strategies were applied: (*eNOS* OR endothelial nitric oxide synthase OR nitric oxide synthase type III OR *NOS3*) AND (polymorphism OR variant OR mutation OR genotype OR allele) AND (diabetes OR mellitus OR diabetes mellitus OR DM). The literature search was updated to 15 March 2022. Furthermore, the reference lists of previously published meta-analyses were carefully reviewed to identify additional eligible studies (Jia et al., 2013; Zhang et al., 2017; Dong et al., 2018).

### Selection Criteria

Inclusion criteria were as listed below: 1) case-control or cohort studies; 2) described the association between the *eNOS* 4b/a, G894T and T786C polymorphisms and risk of T2DM; 3) provided sufficient genotype data or the odds ratio (OR) with 95% confidence intervals (CI) in the selected literature. Exclusion criteria were as listed below: 1) duplicate genotype data; 2) studies with no available data; 3) meta-analyses of case reports, abstracts, reviews and letters.

### Data Extraction and Quality Score Assessment

Two investigators independently extracted the data and cross-examined it, trying to resolve differences through discussion. If no consensus was reached after discussion, the third author would be invited to extract the data again for final review and confirmation. Moreover, the original authors could be contacted via e-mail if necessary. Races were divided into “Caucasians,” “Asians,” “Indians,” and “Africans.” “Mixed populations” was defined if race was not stated or the sample size of several races cannot be separated in original study.

The Newcastle-Ottawa Scale (NOS) (Wells et al., 2014) was applied by the two investigators to independently assess the quality of all included research. These scales are influenced by three factors: selection (four points), comparability (two points), and exposure (three points). Hardy–Weinberg Equilibrium (HWE) was employed to conduct a quality assessment on the basis of NOS (one point). The overall score varied from zero (worst) to ten (highest), with seven points or more as high quality.

### Trial Sequential Analysis

Meta-analyses could increase the power and accuracy of evaluating intervention effects and are regarded as good evidence when available studies are used. However, misleading conclusions may be generated owing to random mistakes if the sample size is very small. Therefore, TSA was carried out to decrease random mistakes and predict the required information size (RIS) in this study (Brok et al., 2008; Thorlund et al., 2011). TSA was performed with the help of TSA 0.9 software (Copenhagen Trial Unit, Centre for Clinical Intervention Research, Copenhagen). The random effect model was used in this work. Alpha (type I error) and beta (type II error) were given as 0.05 and 0.2, respectively. The accruing information size (AIS) was used to determine information size, and the OR value was used to determine the combined effect amount. Based on O'Brien-Fleming-spending functions, a TSA employs trial sequential monitoring boundaries. In addition, the relative risk reduction (RRR) is set at 15% (Kulinskaya and Wood, 2014). If the cumulative Z-curve passes the monitoring border, the RIS line, or enters the futility region, strong evidence for our study may well be affirmed. Otherwise, additional research is required (Wetterslev et al., 2009).

### Statistical Analysis

Potential associations between the *eNOS* genetic polymorphisms (4b/a VNTR, G894T and T786C) and T2DM risk were expressed by ORs and corresponding 95% CIs. Five genetic models were used for comparison: hybrid, homozygous, dominant, recessive and allele model. Chi-square-based Q-test and *I* ^*2*^ value were employed in assessing the Heterogeneity. When *P* was less than 0.10 and/or *I* ^*2*^ was greater than 50% (Li et al., 2005), the random-effects model (Mantel and Haenszel, 1959) was adopted because of the significant heterogeneity. On the contrary, the fixed-effects model (DerSimonian and Laird, 2015) was adopted. In addition, the source of heterogeneity was explored by meta-regression analysis. Subgroups were conducted by race, type of control, age and gender. Three methods were applied for sensitivity analyses: 1) excluded one study in turn; 2) eliminated low-quality and medium-quality or Hardy–Weinberg Disequilibrium (HWD) studies; 3) kept only high-quality and HWE studies. Furthermore, HWE was assessed using the Chi-square goodness-of-fit test. *p* > 0.05 was defined as HWE, otherwise as HWD in the control group. Begg’s funnel plot (Begg and Mazumdar, 1994) and Egger’s test (Egger et al., 1997) were applied to evaluate publication bias. If there were publication bias, the number of missing studies would be estimated and supplemented using a nonparametric “trim and fill” method (Dual and Tweedie, 2000). The false-positive report probability (FPRP) (Wacholder et al., 2004), Bayesian False Discovery Probability (BFDP) (Wakefield, 2007), and the Venice criteria (Ioannidis et al., 2008) were used to evaluate the credibility of statistically significant associations. Stata 12.0 software (STATA Corporation, College Station, TX) was applied to calculate all statistical analyses.

## Results

### Study Characteristics

**FIGURE 1:**
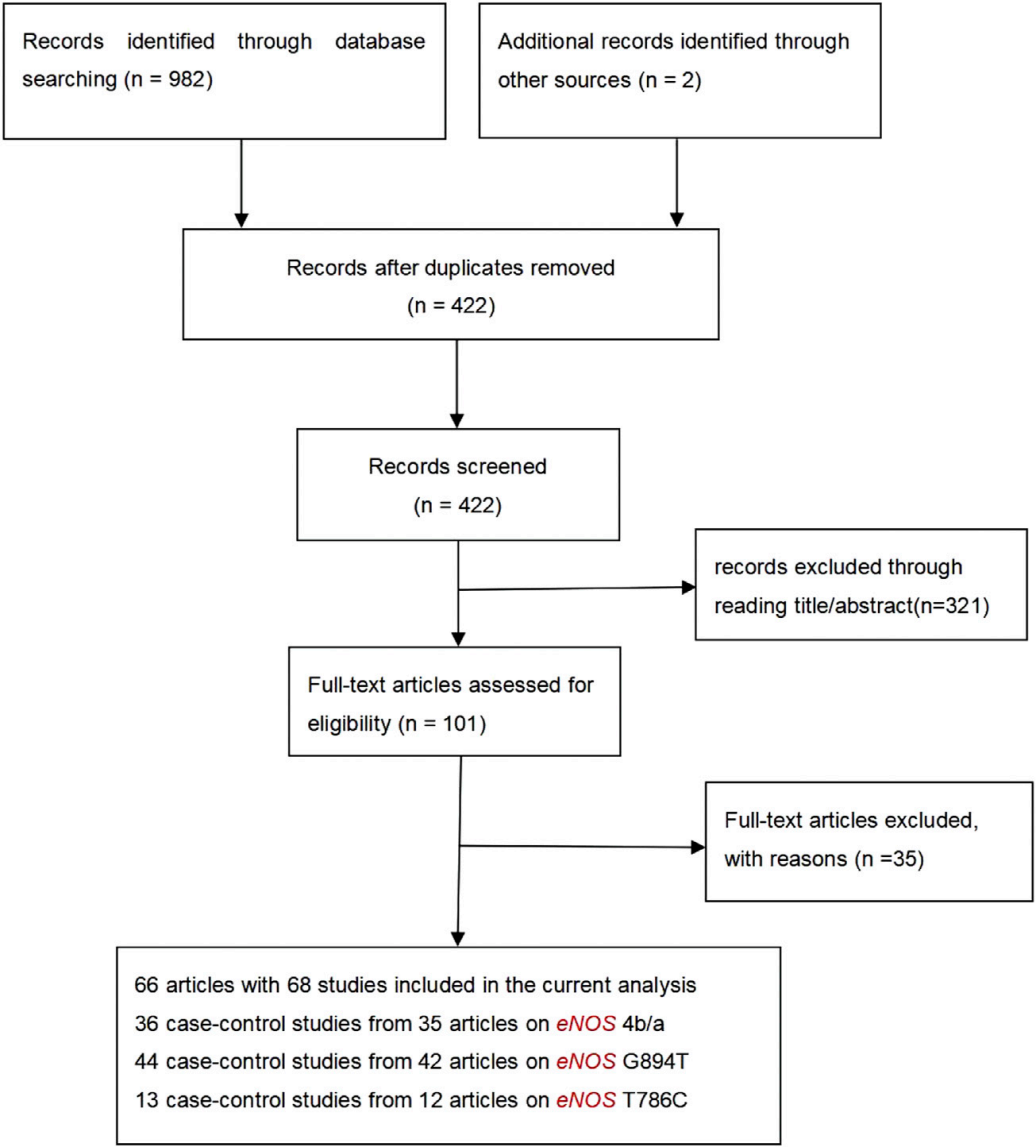
Flow diagram of the literature search.

Initially, 984 articles were retrieved from PubMed, CNKI, Medline, Embase, ISI and the China Wan-fang databases. We excluded 321 papers by carefully evaluating titles and abstracts. Moreover, 16 were excluded due to duplication and invalidation of data, and 19 were excluded due to inadequate controls. Finally, 66 articles with 68 studies were eligible for inclusion in our meta-analysis (Table 1). The detailed investigation process is shown in Figure 1. A total of 68 studies (Figure 1) met our inclusion criteria (involving 15,988 T2DM cases and 25,452 controls), of which 36 studies reported the *eNOS* 4b/a (8,553 cases and 6,613 controls), 44 studies investigated the *eNOS* G894T (10,722 cases and 21,256 controls), and 13 studies reported the *eNOS* T786C (4,676 cases and 3,842 controls), as shown in Tables 2–4. Furthermore, twenty, thirty-seven, six, two and three studies were conducted to investigate Caucasians, Asians, Indians, Africans, and mixed groups, respectively. In addition, the *eNOS* 4b/a had 19 high-quality studies and 17 low-quality studies, the *eNOS* G894T had 18 high-quality studies and 26 low-quality studies, and the *eNOS* T786C had five high-quality studies and eight low-quality studies. Moreover, the complete features, scores, HWE and the genotype frequencies of the selected literature were shown in Tables 2–4. Furthermore, Table 5 showed the results of the detailed quality scores for the included articles according to the NOS.

**TABLE 1:** Included studies of *eNOS* polymorphism in T2DM within the meta-analyses.

First author/Year	Country	Eligible Research Studies of 4b/a				Eligible Research Studies of G894T			Eligible Research Studies of T786C	
		This Study	Dong et al. (2018)	Zhang et al. (2017)	Jia et al. (2013)	This Study	Dong et al. (2018)	Jia et al. (2013)	This Study	Dong et al. (2018)
Wang et al. (1999)	Japan	A	—	A	—	—	—	—	—	—
Pulkkinen et al. (2000)	Finland	C	C	—	C	C	C	C	—	—
Suzuki et al. (2000)	Japan	—	—	—	–	A	A (NOT)	—	—	—
Neugebauer et al. (2000)	Japan	A	A	—	EA	—	—	—	—	—
Ukkola et al. (2001)	Finland	—	—	—	—	C	C	C	–	–
Li et al. (2001)	China	A	A	A	A	A	A	A	—	—
Asakimori et al. (2001)	Japan	A	A	—	—	—	—	—	—	—
Ohtoshi et al. (2002)	Japan	—	—	—	—	A	A	—	A	A
Noiri et al. (2002)	Japan	—	—	—	—	A	A	—	—	—
Lin et al. (2002)	China	A	A	A	EA	—	—	—	—	—
Huang et al. (2002)	China	A	—	—	A	—	—	—	—	—
Monti et al. (2003)	Italy	—	—	—	—	C	C	C	—	—
Ksiazek et al. 2003	Poland	C	C(NOT)	—	—	—	—	—	—	—
Lee et al. (2003)	Taiwan	A	A	—	A	—	—	—	—	—
Luo and Ning, (2003)	China	A	–	A (NOT)	A (NOT)	—	—	—	—	—
Nagase et al. (2003)	Japan	—	—	—	—	A	—	—	—	—
Zhang et al. (2003)	China	A	–	–	A	—	—	—	—	—
Ren et al. (2003)	China	—	—	—	—	A	—	A	—	—
Ma (2003)	China	A	—	—	—	—	—	—	—	—
Sun et al. (2004)	China	A	—	A (NOT)	EA	—	—	A	—	—
Shin Shin et al. (2004)	Korea	—	—	–	—	A	A	A	—	—
Dong et al., 2005	China	A	—	–	—	A	—	—	—	—
Zhang et al., 2005	China	A	—	A	—	—	—	—	—	—
Wang, (2005)	China	A	–	—	–	–	–	–	–	–
Sandrim et al., 2006	Brazil	M	C	—	C	M	C	C	M	C
de Syllos et al., 2006	Brazil	M	C	—	C	M	C	C	M	C
Zheng-ju et al. (2006)	China	—	—	—	–	A	—	—	—	—
Luo et al., 2006	China	—	—	—	–	A	—	A	—	—
Wu et al., 2007	China	A	—	—	A	–	—	—	—	—
Fu et al., 2007	China	—	—	—	—	A	—	A	–	–
Ma et al., 2007	China	—	—	—	—	A	—	A	–	–
Ezzidi et al., 2008	Tunisia	Af	C	–	Af	Af	C	Af	Af	C
Ritt et al., 2008	Germany	—	—	—	—	C	C	C	–	–
Thaha et al., 2008	Japan	—	—	—	—	A	A	—	—	—
Odeberg et al., 2008	Sweden	—	—	—	—	C	C	C	—	—
Galanakis et al., 2008	Greece	C	C(NA)	–	C	—	—	—	—	—
Szabó et al., 2009	Hungary	–	–	—	—	C	C	—	—	—
Kincl et al., 2009	Czech Republic	C	C	—	—	—	—	—	—	—
Deng et al., 2009	China	A	–	—	A (NOT)	A	—	A (NOT)	—	—
Yu et al., 2009	China	A	–	—	A	–	—	–	—	—
Kim et al., 2010	Korea	A	A	—	–	A	A	–	A	A
Corapcioglu et al., 2010	Turkey	–	–	—	–	C	C	–	–	–
Bae et al., 2010	Korea	A	A	—	A	A	A	A	A	A
Li et al., 2010	China	A	–	A (NOT)	–	–	–	–	–	–
Mehrab-Mohseni et al. (2011)	Iran	C	—	—	A	–	–	–	–	–
El-Din Bessa and Hamdy, (2011)	Egypt	—	—	—	–	C	C	–	–	–
Angeline et al., 2011	India	—	—	—	–	–	C(NOT)	A	–	–
Santos et al., 2011	Brazil	M	C	—	C	M	C	C	M	C
Guo and Liu, (2012)	China	A	–	—	—	—	—	—	—	—
Li et al., 2011	China	—	—	—	—	A	—	—	—	—
Hou et al., 2012	China	—	—	—	—	A	A	—	—	—
Dai and Zhang, (2012)	China	—	—	—	—	A	—	—	—	—
Bressler et al., 2013	America	—	—	—	—	C	C	—	—	—
Rahimi et al., 2013	Iran	C	A	—	—	—	—	—	—	—
Jamil et al., 2014	India	–	–	—	—	—	A	—	—	—
Mackawy et al., 2014	Saudi Arabia	–	–	—	—	C	C	—	—	—
Li et al., 2015	China	–	–	—	—	A	A	–	A	A
Haldar et al., 2015	India	–	–	—	—	–	—	—	—	A
She et al., 2015	China	A	A	—	—	–	—	—	—	—
Momeni et al., 2016	Iran	—	—	—	—	C	—	—	—	—
Moguib et al., 2017	Egypt	—	—	—	–	C	—	—	**C**	—
Rizvi et al., 2019	India	—	—	—	–	–	—	—	—	—
Yigit et al., 2020	Turkey	C	—	—	–	–	—	—	—	—
Abdullah et al. (2021)	Jordan	C	—	—	–	C	—	—	C	–
Raina et al., 2021	India	—	—	—	—	—	—	—	—	—
Gusti et al., 2021	Saudi Arabia	—	—	—	—	C	—	—	—	—

A, asian; I, indian; Af, African; E, european; C, caucasian; Ar: Arabs; M, mixed; U, unidentified; EA: East Asian; SA: South-Asian; WA:West-Asian; HWE YES:*p* > 0.05; NOT:*p* < 0.05; NA:not available.

**TABLE 2:** Genotype distribution of *eNOS* 4b/a polymorphisms in the included studies of T2DM.

First author/Year	Ethnicity	Sample Size	Matching	Type of Control	Case			Control			HWE (P)	Quality Score
					aa	ab	bb	aa	ab	bb		
Wang et al. (1999)	Asian	71/248	Age and sex	Healthy controls	0	13	58	0	47	201	0.099	8
Pulkkinen et al. (2000)	Caucasian	251/110	NR	Non-diabetic controls	11	85	155	5	26	79	0.152	9
Neugebauer et al. (2000)	Asian	215/155	Age and sex	Healthy controls	7	36	172	0	22	133	0.342	7
Li et al. (2001)	Asian	143/85	Age and sex	Healthy controls	0	40	103	1	22	62	0.535	8
Asakimori et al. (2001)	Asian	295/189	Age and sex	Non-diabetic controls	3	67	225	0	26	163	0.31	7
Huang et al. (2002)	Asian	85/68	Age and sex	Non-diabetic controls	3	16	66	0	7	61	0.655	7
Lin et al. (2002)	Asian	127/70	NR	Healthy controls	1	14	112	0	6	64	0.780	4
Ksiazek et al. (2003)	Caucasian	410/330	Age and sex	Healthy controls	34	124	252	4	74	252	0.580	8
Lee et al. (2003)	Asian	800/398	Age and sex	Healthy controls	14	112	674	1	57	340	0.386	8
Luo and Ning, (2003)	Asian	84/37	Age	Healthy controls	35	6	43	2	1	34	0.000	6
Zhang et al., 2003	Asian	132/80	Age and sex	Healthy controls	2	19	111	0	12	68	0.468	8
Ma, (2003)	Asian	299/100	Age and sex	Healthy controls	3	64	232	0	18	82	0.323	8
Sun et al., 2004	Asian	399/113	Age and sex	Healthy controls	6	22	311	2	18	93	0.320	8
Dong et al., 2005	Asian	134/85	Age and sex	Healthy controls	0	38	96	0	22	62	0.167	6
Zhang et al., 2005	Asian	322/166	Age and sex	Healthy controls	2	42	278	1	20	145	0.734	8
Wang. (2005)	Asian	204/100	Age and sex	Healthy controls	0	49	155	0	13	87	0.487	9
Sandrim et al. (2006)	Mixed	66/102	Age and sex	Healthy controls	2	16	48	4	19	79	0.056	7
de Syllos et al. (2006)	Mixed	170/103	Age and sex	Healthy controls	5	43	122	4	20	79	0.079	7
Wu et al. (2007)	Asian	80/119	Age and sex	Healthy controls	0	13	67	0	23	86	0.218	9
Ezzidi et al. (2008)	African	917/748	Age and sex	Healthy controls	50	305	548	20	217	511	0.594	7
Galanakis et al. (2008)	Caucasian	108/160	NR	Healthy controls	5	29	74	1	39	120	0.250	5
Kincl et al. (2009)	Caucasian	348/813	NR	Non-diabetic controls	12	107	229	32	228	553	0.169	5
Deng et al. (2009)	Asian	108/100	Age and sex	Healthy controls	3	19	86	9	16	75	0.000	7
Yu et al. (2009)	Asian	76/100	NR	Non-diabetic controls	2	10	64	1	12	87	0.433	6
Kim et al. (2010)	Asian	36/170	Age and sex	Non-diabetic controls	0	12	24	0	26	144	0.280	6
Bae et al. (2010)	Asian	89/299	Age and sex	Non-diabetic controls	2	26	61	0	51	248	0.107	8
Li et al. (2010)	Asian	166/85	Age and sex	Non-diabetic controls	12	24	130	3	14	68	0.057	8
Mehrab-Mohseni et al. (2011)	Caucasian	220/96	Age and sex	Healthy controls	9	54	157	0	16	80	0.373	9
Santos et al. (2011)	Mixed	617/100	Age and sex	Healthy controls	16	158	405	3	30	67	0.871	9
Guo and Liu, (2012)	Asian	144/63	Age and sex	Healthy controls	9	7	126	2	1	60	0.000	6
Rahimi et al. (2013)	Caucasian	173/101	Age	Healthy controls	3	46	124	0	28	73	0.106	8
She et al. (2015)	Asian	278/223	Age and sex	Non-diabetic controls	1	60	217	1	24	198	0.768	8
Yigit et al. (2020)	Caucasian	85/282	Age and sex	Non-diabetic controls	2	12	71	11	115	156	0.068	8
Abdullah et al. (2021)	Caucasian	103/100	Age and sex	Healthy controls	3	31	69	3	27	70	0.840	6
Raina et al. (2021)	Indian	461/315	Age and sex	Healthy controls	17	137	307	6	95	214	0.217	8
Raina et al. (2021)	Indian	337/200	Age and sex	Healthy controls	12	110	215	5	30	145	0.036	7

HWE, Hardy–Weinberg equilibrium; *eNOS*, endothelial nitric oxide synthase; NR, not reported; NA, not available.

**TABLE 3:** Genotype distribution of *eNOS* G894T polymorphisms in the included studies of T2DM.

First author/Year	Ethnicity	Sample Size	Matching	Type of Control	Case			Control			HWE (P)	Quality Score
					GG	GT	TT	GG	GT	TT		
Pulkkinen et al. (2000)	Caucasian	251/110	NR	Non-diabetic controls	136	97	18	54	45	11	0.720	9
Suzuki et al. (2000)	Asian	48/270	Age and sex	Healthy controls	38	8	2	250	18	2	0.016	7
Ukkola et al., 2001	Caucasian	216/222	Age and sex	Healthy controls	106	93	17	112	92	18	0.883	7
Li et al., 2001	Asian	143/85	Age and sex	Healthy controls	93	49	1	63	21	1	0.606	8
Ohtoshi et al., 2002	Asian	301/233	Age and sex	Healthy controls	256	42	3	196	35	2	0.753	7
Noiri et al., 2002	Asian	72/304	Age and sex	Healthy controls	49	23	0	251	53	0	0.096	7
Monti et al., 2003	Caucasian	159/207	NR	Healthy controls	52	63	44	86	82	29	0.199	6
Nagase et al., 2003	Asian	71/248	Age and sex	Healthy controls	38	8	2	250	18	2	0.016	7
Ren et al., 2003	Asian	211/83	Age and sex	Healthy controls	159	28	3	67	15	0	0.188	8
Shin Shin et al. (2004)	Asian	177/129	Age and sex	Non-diabetic controls	147	30	0	116	13	0	0.547	9
Dong et al. 2005	Asian	134/85	Age and sex	Healthy controls	88	45	1	63	21	1	0.606	6
Sandrim et al. 2006	Mixed	66/102	Age and sex	Healthy controls	34	28	4	53	45	4	0.138	7
de Syllos et al. 2006	Mixed	170/103	Age and sex	Healthy controls	82	78	10	54	45	4	0.146	7
Zheng-ju et al. (2006)	Asian	136/61	Age and sex	Healthy controls	95	41	0	49	12	0	0.394	5
Luo et al., 2006	Asian	80/119	Age and sex	Healthy controls	63	17	0	98	19	2	0.351	9
Fu et al., 2007	Asian	139/63	Age and sex	Healthy controls	97	42	0	51	12	0	0.403	8
Ma et al., 2007	Asian	299/100	Age and sex	Healthy controls	240	59	0	86	14	0	0.452	8
Ezzidi et al., 2008	African	917/748	Age and sex	Healthy controls	350	442	122	335	334	69	0.274	7
Ritt et al., 2008	Caucasian	84/84	Age and sex	Non-diabetic controls	37	47	0	38	46	0	0.001	8
Thaha et al., 2008	Asian	39/100	Age and sex	Healthy controls	7	31	1	72	26	2	0.845	7
Odeberg et al., 2008	Caucasian	403/799	Age and sex	Non-diabetic controls	222	153	28	407	322	70	0.580	9
Szabó et al., 2009	Caucasian	209/384	Sex	Healthy controls	87	92	30	201	161	22	0.162	6
Deng et al., 2009	Asian	108/100	Age and sex	Healthy controls	83	20	5	80	14	6	0.000	7
Kim et al., 2010	Asian	36/170	Age and sex	Non-diabetic controls	33	3	0	135	35	0	0.135	6
Corapcioglu et al., 2010	Caucasian	97/102	Age and sex	Healthy controls	46	46	5	48	42	12	0.549	8
Bae et al., 2010	Asian	89/299	Age and sex	Non-diabetic controls	75	14	0	245	53	1	0.290	8
El-Din Bessa and Hamdy, (2011)	Caucasian	80/20	Age and sex	Healthy controls	27	37	16	12	7	1	0.987	7
Angeline et al., 2011	Indian	100/160	Age and sex	Non-diabetic controls	25	55	20	113	47	0	0.029	7
Santos et al., 2011	Mixed	617/100	Age and sex	Healthy controls	294	261	54	47	48	5	0.098	9
Li et al., 2011	Asian	326/215	Age and sex	Healthy controls	258	67	1	171	33	1	0.659	8
Hou et al., 2012	Asian	100/50	Age and sex	Healthy controls	12	63	25	12	25	13	0.998	6
Dai and Zhang, (2012)	Asian	120/60	Age and sex	Healthy controls	75	45	0	43	17	0	0.201	6
Bressler et al., 2013	Caucasian	980/9,657	Age and sex	Non-diabetic controls	450	426	104	4,506	4,181	970	0.998	8
Bressler et al., 2013	African	728/3,009	Age and sex	Non-diabetic controls	580	139	9	2,338	626	45	0.676	8
Jamil et al., 2014	Indian	196/190	Age and sex	Healthy controls	109	88	0	162	28	0	0.273	7
Mackawy et al., 2014	Caucasian	80/40	Age and sex	Non-diabetic controls	31	32	17	19	16	5	0.576	7
Li et al., 2015	Asian	1,234/1,272	Age and sex	Healthy controls	1,024	189	3	978	257	9	0.074	7
Momeni et al., 2016	Caucasian	94/94	Age and sex	Non-diabetic controls	3	33	58	10	22	62	0.002	6
Moguib et al., 2017	Caucasian	200/100	Age and sex	Healthy controls	122	64	14	46	52	2	0.004	6
Rizvi et al., 2019	Indian	200/200	Age and sex	Healthy controls	133	57	10	132	54	14	0.015	6
Abdullah et al. (2021)	Caucasian	103/100	Age and sex	Healthy controls	59	35	9	49	40	11	0.516	6
Raina et al., 2021	Indian	461/315	Age and sex	Healthy controls	289	159	13	214	96	5	0.115	8
Raina et al., 2021	Indian	337/200	Age and sex	Healthy controls	192	133	12	137	58	5	0.696	8
Gusti et al., 2021	Caucasian	111/164	Age and sex	Non-diabetic controls	63	39	9	107	51	6	0.980	8

HWE, Hardy–Weinberg equilibrium; *eNOS*, endothelial nitric oxide synthase; NR, not reported; NA, not available.

**TABLE 4:** Genotype distribution of *eNOS* T786C polymorphisms in the included studies of T2DM.

First author/Year	Ethnicity	Sample Size	Matching	Type of Control	Case			Control			HWE (P)	Quality Score
					TT	TC	CC	TT	TC	CC		
Ohtoshi et al., 2002	Asian	301/233	Age and sex	Healthy controls	250	48	3	194	35	4	0.115	7
Sandrim et al., 2006	Mixed	66/102	Age and sex	Healthy controls	34	28	4	38	52	12	0.361	7
de Syllos et al., 2006	Mixed	170/103	Age and sex	Healthy controls	77	78	15	38	53	12	0.314	7
Ezzidi et al., 2008	African	917/748	Age and sex	Healthy controls	485	354	66	436	264	36	0.623	7
Kim et al., 2010	Asian	36/170	Age and sex	Non-diabetic controls	26	10	0	145	25	0	0.301	6
Bae et al., 2010	Asian	89/299	Age and sex	Non-diabetic controls	63	24	2	250	49	0	0.123	8
Santos et al., 2011	Mixed	617/100	Age and sex	Healthy controls	233	264	120	42	46	12	0.913	9
Li et al., 2015	Asian	1,234/1,272	Age and sex	Healthy controls	916	268	20	960	264	16	0.653	7
Haldar et al., 2015	Indian	145/100	Age and sex	Healthy controls	80	50	15	84	14	2	0.146	8
Moguib et al., 2017	Caucasian	200/100	Age and sex	Healthy controls	48	152	0	33	67	0	0.000	6
Abdullah et al. (2021)	Caucasian	103/100	Age and sex	Healthy controls	45	45	13	41	42	17	0.277	6
Raina et al., 2021	Indian	461/315	Age and sex	Healthy controls	273	177	11	220	90	5	0.215	8
Raina et al., 2021	Indian	337/200	Age and sex	Healthy controls	210	116	11	145	50	5	0.782	8

HWE, Hardy–Weinberg equilibrium; *eNOS*, endothelial nitric oxide synthase; NR, not reported; NA, not available.

**TABLE 5:** Quality assessment of included studies based on the Newcastle-Ottawa Scale for assessing the quality of case control studies.

First Author/Year	Selection				Comparability		Exposure			HWE (p)	Quality Score (Total score)
	Adequate Definition of Case	Representativeness of the Cases	Selection of Controls	Definition of Controls	Age and Sex	Any Additional Factor	Ascertainment of Exposure	Same Method of Ascertainment for Cases and Controls	Non-Response Rate		
Wang et al. (1999)	0	1	1	1	1	1	1	1	0	1	8
Pulkkinen et al. (2000)	1	1	1	1	0	1	1	1	1	1	9
Suzuki et al. (2000)	0	1	1	1	1	1	1	1	0	0	7
Neugebauer et al. (2000)	0	1	0	1	1	1	1	1	0	1	7
Ukkola et al. 2001	0	1	1	1	1	0	1	1	0	1	7
Li et al. 2001	1	1	0	1	1	1	1	1	0	1	8
Asakimori et al., 2001	0	1	0	1	1	1	1	1	0	1	7
Ohtoshi et al. 2002	1	1	0	1	1	0	1	1	0	1	7
Noiri et al. 2002	0	1	0	1	1	1	1	1	0	1	7
Lin et al. 2002	1	1	0	1	1	0	1	1	0	1	7
Huang et al. 2002	0	1	0	1	0	0	0	1	0	1	4
Monti et al. 2003	0	1	1	1	0	0	1	1	0	1	6
Ksiazek et al. 2003	1	1	1	1	1	0	1	1	0	1	8
Lee et al., 2003	1	1	0	1	1	1	1	1	0	1	8
Luo and Ning, (2003)	1	1	0	1	1	0	1	1	0	0	6
Nagase et al. 2003	0	1	1	1	1	1	1	1	0	0	7
Zhang et al. 2003	1	1	1	1	1	0	1	1	0	1	8
Ren et al. 2003	1	1	0	1	1	1	1	1	0	1	8
Ma, (2003)	1	1	0	1	1	1	1	1	0	1	8
Sun et al., 2004	1	1	1	1	1	0	1	1	0	1	8
Shin Shin et al. (2004)	1	1	1	1	1	1	1	1	0	1	9
Dong et al. 2005	0	1	0	1	1	0	1	1	0	1	6
Zhang et al. 2005	1	1	1	1	1	0	1	1	0	1	8
Wang (2005)	1	1	1	1	1	1	1	1	0	1	9
Sandrim et al. 2006	0	1	1	1	1	0	1	1	0	1	7
de Syllos et al. 2006	0	1	1	1	1	0	1	1	0	1	7
Zheng-ju et al. (2006)	0	1	0	1	1	0	0	1	0	1	5
Luo et al., 2006	1	1	1	1	1	1	1	1	0	1	9
Wu et al., 2007	1	1	1	1	1	1	1	1	0	1	9
Fu et al., 2007	1	1	0	1	1	1	1	1	0	1	8
Ma et al., 2007	1	1	0	1	1	1	1	1	0	1	8
Ezzidi et al., 2008	0	1	0	1	1	1	1	1	0	1	7
Ritt et al., 2008	1	1	1	1	1	1	1	1	0	0	8
Thaha et al., 2008	0	1	0	1	1	1	1	1	0	1	7
Odeberg et al., 2008	1	1	1	1	1	1	1	1	0	1	9
Galanakis et al., 2008	0	1	0	1	0	0	1	1	0	1	5
Szabó et al., 2009	0	1	0	1	1	0	1	1	0	1	6
Kincl et al., 2009	0	1	0	1	0	0	1	1	0	1	5
Deng et al., 2009	1	1	0	1	1	1	1	1	0	0	7
Yu et al., 2009	1	1	0	1	0	0	1	1	0	1	6
Kim et al., 2010	0	1	0	1	1	0	1	1	0	1	6
Corapcioglu et al., 2010	1	1	0	1	1	1	1	1	0	1	8
Bae et al., 2010	1	1	1	1	1	0	1	1	0	1	8
Li et al., 2010	1	1	0	1	1	1	1	1	0	1	8
Mehrab-Mohseni et al. (2011)	1	1	1	1	1	1	1	1	0	1	9
El-Din Bessa and Hamdy, (2011)	1	1	0	1	1	0	1	1	0	1	7
Angeline et al., 2011	1	1	1	1	1	0	1	1	0	0	7
Santos et al., 2011	1	1	1	1	1	1	1	1	0	1	9
Guo and Liu, (2012)	1	1	0	1	1	0	1	1	0	0	6
Li et al., 2011	1	1	0	1	1	1	1	1	0	1	8
Hou et al., 2012	0	1	0	1	1	0	1	1	0	1	6
Dai and Zhang, (2012)	0	1	0	1	1	0	1	1	0	1	6
Bressler et al., 2013	0	1	1	1	1	1	1	1	0	1	8
Rahimi et al., 2013	1	1	0	1	1	1	1	1	0	1	8
Jamil et al., 2014	0	1	0	1	1	1	1	1	0	1	7
Mackawy et al., 2014	1	1	0	1	1	0	1	1	0	1	7
Li et al., 2015	1	1	0	1	1	0	1	1	0	1	7
Haldar et al., 2015	1	1	0	1	1	1	1	1	0	1	8
She et al., 2015	0	1	1	1	1	1	1	1	0	1	8
Momeni et al., 2016	1	1	0	1	1	0	1	1	0	0	6
Moguib et al., 2017	0	1	1	1	1	0	1	1	0	0	6
Rizvi et al., 2019	1	1	0	1	1	0	1	1	0	0	6
Yigit et al., 2020	1	1	0	1	1	1	1	1	0	1	8
Abdullah et al. (2021)	0	1	0	1	1	0	1	1	0	1	6
Raina et al., 2021	1	1	1	1	1	0	1	1	0	1	8
Gusti et al., 2021	0	1	0	1	1	1	1	1	0	1	8

HWE, Hardy–Weinberg equilibrium.

### Quantitative Synthes

**FIGURE 2:**
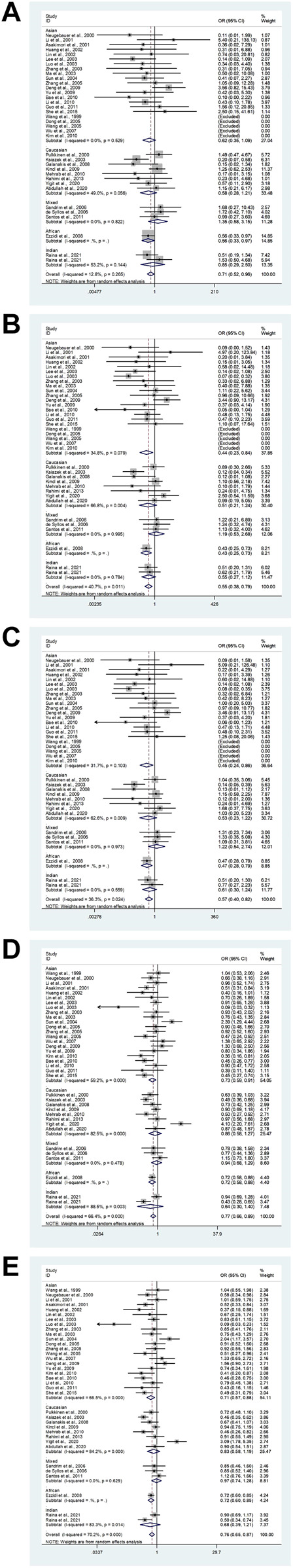
The forest plots of all selected studies on the association between eNOS 4b/a polymorphism and the risk of T2DM in different races [**(A)**: hybrid model; **(B)** homozygous model; **(C)** dominant model; **(D)** recessive model; **(E)** allele model].

In the total analysis, the *eNOS* 4b/4a was associated with a substantially lower T2DM risk (ab vs. aa: OR = 0.71, 95% CI = 0.52–0.96; bb vs. aa: OR = 0.55, 95% CI = 0.38–0.79; ab + bb vs. aa: OR = 0.58, 95% CI = 0.40–0.82; bb vs. aa + ab: OR = 0.77, 95% CI = 0.66–0.89; b vs. a: OR = 0.76, 95% CI = 0.65–0.87, Table 6; Figure 2). In the following ethnic subgroup analysis, we discovered a significant association between *eNOS* 4b/4a polymorphism and T2DM susceptibility in the Asian population (bb vs. aa: OR = 0.44, 95% CI = 0.23–0.84; ab + bb vs. aa: OR = 0.45, 95% CI = 0.24–0.86; bb vs. aa + ab: OR = 0.73, 95% CI = 0.59–0.91; b vs. a: OR = 0.71, 95% CI = 0.57–0.88, Table 6; Figure 2). Also, similar association was also found in the healthy control and matched studies (Table 6).

**TABLE 6:** Meta-analysis of the association of *eNOS* 4b/4a polymorphism with risk of T2DM.

Variable	n (Cases/Controls)	ab vs. aa		bb vs. aa		ab + bb vs. aa		bb vs. aa + ab		b vs. a	
		Or (95%CI)	*P*_h_/*I* ^2^ (%)	Or (95%CI)	*P*_h_/*I* ^2^ (%)	Or (95%CI)	*P*_h_/*I* ^2^ (%)	Or (95%CI)	*P*_h_/*I* ^2^ (%)	Or (95%CI)	*P*_h_/*I* ^2^ (%)
Overall	36 (8,553/6,613)	**0.71 (0.52–0.96)**	0.265/12.8	**0.5**5 (0.38–0.79)	0.011/40.7	**0.58 (0.40–0.82)**	0.024/36.3	**0.77 (0.66–0.89)**	<0.001/66.4	**0.76 (0.65–0.87)**	<0.001/70.2
Ethnicity											
Asian	22 (4,287/3,053)	0.62 (0.35–1.09)	0.529/0.0	**0.44 (0.23–0.84)**	0.079/34.8	**0.45 (0.24–0.86)**	0.103/31.7	**0.73 (0.59–0.91)**	<0.001/59.2	**0.71 (0.57–0.88)**	<0.001/66.5
Caucasian	8 (1,698/1992)	0.58 (0.28–1.21)	0.056/49.0	0.51 (0.21–1.24)	0.004/66.8	0.53 (0.23–1.22)	0.009/62.6	0.86 (0.58–1.27)	<0.001/82.5	0.83 (0.58–1.19)	<0.001/84.2
Type of control											
Healthy controls	26 (6,844/4,274)	**0.66 (0.44**–**0.98)**	0.195/20.1	**0.50 (0.32**–**0.78)**	0.016/43.5	**0.52 (0.34**–**0.82)**	0.024/41.0	**0.79 (0.67**–**0.93)**	<0.001/56.7	**0.77 (0.66**–**0.91)**	<0.001/66.3
Non-diabetic controls	10 (1709/2,339)	0.92 (0.57–1.49)	0.589/0.0	0.78 (0.45–1.36)	0.318/14.0	0.86 (0.54–1.36)	0.440/0.0	0.70 (0.47–1.04)	<0.001/80.6	**0.70 (0.50**–**0.99)**	<0.001/79.4
Matching											
Age and sex	28 (6,902/5,154)	**0.67 (0.46**–**0.97)**	0.226/17.4	**0.59 (0.39**–**0.91)**	0.037/37.6	**0.62 (0.42**–**0.91)**	0.082/30.7	**0.80 (0.67**–**0.95)**	<0.001/69.5	**0.79 (0.67**–**0.93)**	<0.001/70.3
NR	6 (995/1,321)	1.02 (0.59–1.77)	0.439/0.0	0.75 (0.41–1.38)	0.364/8.1	0.81 (0.44–1.47)	0.361/8.6	**0.78 (0.64**–**0.96)**	0.550/0.0	**0.77 (0.63**–**0.96)**	0.306/16.7
**Sensitivity analysis**											
**HWE**											
Overall	32 (7,880/6,213)	**0.64 (0.48–0.85)**	0.423/2.7	**0.55 (0.38–0.80)**	0.092/27.7	**0.58 (0.41–0.81)**	0.167/20.8	**0.80 (0.69–0.93)**	<0.001/62.2	**0.79 (0.69–0.90)**	<0.001/63.7
Ethnicity											
Asian	19 (3,951/2,853)	**0.43 (0.22–0.82)**	0.874/0.0	**0.43 (0.23–0.80)**	0.713/0.0	**0.43 (0.23**–**0.81)**	0.761/0.0	**0.75 (0.62–0.93)**	0.005/51.3	**0.74 (0.62–0.89)**	0.009/48.8
Caucasian	8 (1,698/1992)	0.58 (0.28–1.21)	0.056/49.0	0.51 (0.21–1.24)	0.004/66.8	0.53 (0.23–1.22)	0.009/62.6	0.86 (0.58–1.27)	<0.001/82.5	0.83 (0.58–1.19)	<0.001/84.2
Type of control											
Healthy controls	22 (6,170/3,874)	**0.54 (0.38**–**0.75)**	0.460/0.0	**0.48 (0.31**–**0.74)**	0.174/23.7	**0.50 (0.33**–**0.75)**	0.232/18.5	**0.83 (0.72**–**0.96)**	0.027/40.4	**0.81 (0.71**–**0.93)**	0.004/49.9
Non-diabetic controls	10 (1709/2,339)	0.92 (0.57–1.49)	0.589/0.0	0.78 (0.45–1.36)	0.318/14.0	0.86 (0.54–1.36)	0.440/0.0	0.70 (0.47–1.04)	<0.001/80.6	**0.70 (0.50**–**0.99)**	<0.001/79.4
Matching											
Age and sex	25 (6,313/4,791)	**0.55 (0.40**–**0.76)**	0.545/0.0	**0.53 (0.34**–**0.83)**	0.089/31.4	**0.54 (0.36**–**0.81)**	0.192/21.2	**0.81 (0.68**–**0.98)**	<0.001/69.0	**0.80 (0.68**–**0.94)**	<0.001/69.5
NR	6 (995/1,321)	1.02 (0.59–1.77)	0.439/0.0	0.75 (0.41–1.38)	0.364/8.1	0.81 (0.44–1.47)	0.361/8.6	**0.78 (0.64**–**0.96)**	0.550/0.0	**0.77 (0.63**–**0.96)**	0.306/16.7
**Quality score >7**											
Overall	19 (5,200/3,350)	**0.52 (0.34–0.79)**	0.462/0.0	**0.53 (0.30–0.92)**	0.075/36.1	**0.53 (0.32–0.88)**	0.158/26.4	0.88 (0.69–1.11)	<0.001/74.3	0.85 (0.68–1.05)	<0.001/74.5
Ethnicity											
Asian	12 (2,983/2016)	**0.47 (0.22–0.99)**	0.603/0.0	0.52 (0.26–1.07)	0.494/0.0	0.52 (0.26–1.07)	0.534/0.0	0.85 (0.65–1.10)	0.004/60.4	0.83 (0.65–1.05)	0.008/56.7
Caucasian	5 (1,139/919)	0.45 (0.16–1.23)	0.117/45.8	0.42 (0.11–1.65)	0.007/71.5	0.43 (0.13–1.48)	0.020/65.7	0.89 (0.45–1.78)	<0.001/89.6	0.84 (0.45–1.55)	<0.001/89.9
Type of control											
Healthy controls	14 (4,331/2,351)	**0.42 (0.25–0.70)**	0.586/0.0	**0.43 (0.22–0.85)**	0.131/33.5	**0.43 (0.23–0.81)**	0.208/24.7	0.88 (0.71–1.10)	0.002/59.9	0.85 (0.69–1.06)	<0.001/65.9
Non-diabetic controls	5 (869/999)	0.77 (0.35–1.69)	0.354/9.1	0.77 (0.31–1.92)	0.198/33.6	0.78 (0.36–1.71)	0.314/15.7	0.85 (0.40–1.81)	<0.001/89.3	0.82 (0.44–1.55)	<0.001/87.8
Matching											
Age and sex	17 (4,776/3,139)	**0.44 (0.28–0.70)**	0.630/0.0	**0.51 (0.27–0.95)**	0.055/41.0	**0.49 (0.28–0.87)**	0.148/28.8	0.89 (0.69–1.15)	<0.001/76.6	0.85 (0.67–1.08)	<0.001/77.1
**HWE and Quality score > 7**											
Overall	19 (5,200/3,350)	**0.52 (0.34–0.79)**	0.462/0.0	**0.53 (0.30–0.92)**	0.075/36.1	**0.53 (0.32–0.88)**	0.158/26.4	0.88 (0.69–1.11)	<0.001/74.3	0.85 (0.68–1.05)	<0.001/74.5
Ethnicity											
Asian	12 (2,983/2016)	**0.47 (0.22–0.99)**	0.603/0.0	0.52 (0.26–1.07)	0.494/0.0	0.52 (0.26–1.07)	0.534/0.0	0.85 (0.65–1.10)	0.004/60.4	0.83 (0.65–1.05)	0.008/56.7
Caucasian	5 (1,139/919)	0.45 (0.16–1.23)	0.117/45.8	0.42 (0.11–1.65)	0.007/71.5	0.43 (0.13–1.48)	0.020/65.7	0.89 (0.45–1.78)	<0.001/89.6	0.84 (0.45–1.55)	<0.001/89.9
Type of control											
Healthy controls	14 (4,331/2,351)	**0.42 (0.25–0.70)**	0.586/0.0	**0.43 (0.22–0.85)**	0.131/33.5	**0.43 (0.23–0.81)**	0.208/24.7	0.88 (0.71–1.10)	0.002/59.9	0.85 (0.69–1.06)	<0.001/65.9
Non-diabetic controls	5 (869/999)	0.77 (0.35–1.69)	0.354/9.1	0.77 (0.31–1.92)	0.198/33.6	0.78 (0.36–1.71)	0.314/15.7	0.85 (0.40–1.81)	<0.001/89.3	0.82 (0.44–1.55)	<0.001/87.8
Matching											
Age and sex	17 (4,776/3,139)	**0.44 (0.28–0.70)**	0.630/0.0	**0.51 (0.27–0.95)**	0.055/41.0	**0.49 (0.28–0.87)**	0.148/28.8	0.89 (0.69–1.15)	<0.001/76.6	0.85 (0.67–1.08)	<0.001/77.1
**Egger’s test**											
***P***_***E***_		0.381		0.419		0.343		0.871		0.782	

HWE, Hardy–Weinberg equilibrium; *eNOS*, endothelial nitric oxide synthase. The bold values in table indicated that these results are statistically significant.

**FIGURE 3:**
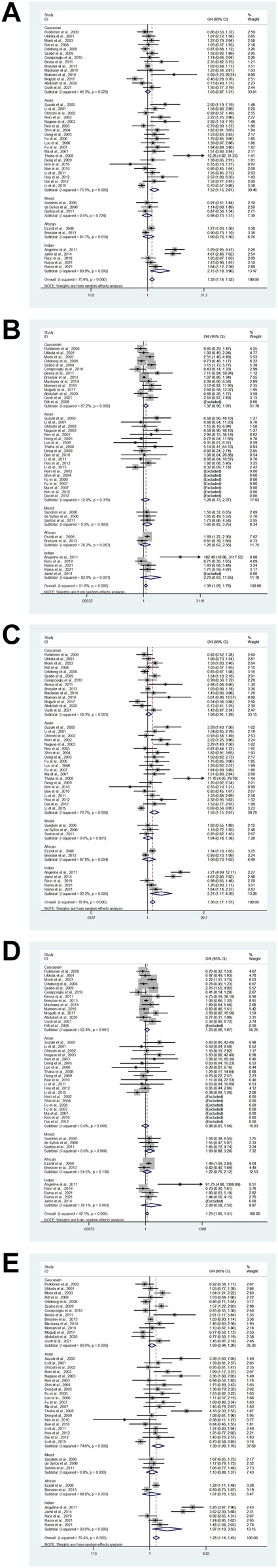
The forest plots of all selected studies on the association between eNOS G894T polymorphism and the risk of T2DM in different races [**(A)**: hybrid model; **(B)** homozygous model; **(C)** dominant model; **(D)** recessive model; **(E)** allele model].

Overall, a substantial association was found between the *eNOS* G894T polymorphism and an increased risk of T2DM (GT vs. GG: OR = 1.32, 95% CI = 1.14–1.52; TT vs. GG: OR = 1.39, 95% CI = 1.09–1.78; GT + TT vs. GG: OR = 1.36, 95% CI = 1.17–1.57; TT vs. GG + GT: OR = 1.23, 95% CI = 1.00–1.51; T vs. G: OR = 1.29, 95% CI = 1.14–1.45, Table 7; Figure 3). Moreover, a significantly increased risk of T2DM was also found in Asians (GT vs. GG: OR = 1.52, 95% CI = 1.15–2.01; GT + TT vs. GG: OR = 1.52, 95% CI = 1.15–2.01; T vs. G: OR = 1.39, 95% CI = 1.09–1.45) and Indians (GT vs. GG: OR = 2.15, 95% CI = 1.18–3.90; GT + TT vs. GG: OR = 2.27, 95% CI = 1.17–4.39; T vs. G: OR = 1.97, 95% CI = 1.10–3.55, Table 7; Figure 3). Furthermore, similar results were also observed in the healthy control and matched analyses (Table 7).

**TABLE 7:** Meta-analysis of the association of *eNOS* G894T polymorphism with risk of T2DM.

Variable	n (Cases/Controls)	GT vs. GG		TT vs. GG		(GT + TT) vs. GG		TT vs. (GG + GT)		T vs. G	
		Or (95%CI)	*P*_h_/*I* ^2^ (%)	Or (95%CI)	*P*_h_/*I* ^2^ (%)	Or (95%CI)	*P*_h_/*I* ^2^ (%)	Or (95%CI)	*P*_h_/*I* ^2^ (%)	Or (95%CI)	*P*_h_/*I* ^2^ (%)
Overall	44 (10722/21256)	**1.32 (1.14–1.52)**	<0.001/75.9	**1.39 (1.09–1.78)**	<0.001/51.8	**1.36 (1.17–1.57)**	<0.001/78.9	**1.23 (1.00–1.51)**	0.005/42.1	**1.29 (1.14–1.45)**	<0.001/79.4
Ethnicity											
Asian	20 (3,863/4,046)	**1.52 (1.15–2.01)**	<0.001/75.5	1.28 (0.73–2.27)	0.315/12.9	**1.52 (1.15–2.01)**	<0.001/76.7	0.98 (0.61–1.56)	0.569/0.0	**1.39 (1.09–1.76)**	<0.001/74.6
Caucasian	14 (3,067/12083)	1.03 (0.87–1.21)	0.029/46.3	1.37 (0.96–1.97)	<0.001/67.2	1.08 (0.91–1.29)	0.003/58.3	1.23 (0.90–1.67)	0.001/62.4	1.09 (0.94–1.26)	<0.001/66.0
Indian	5 (1,294/1,065)	**2.15 (1.18–3.90)**	<0.001/89.9	2.70 (0.63–11.65)	0.001/82.9	**2.27 (1.17–4.39)**	<0.001/92.2	2.08 (0.58–7.52)	0.003/78.1	**1.97 (1.10–3.55)**	<0.001/93.0
Type of control											
HC	32 (7,589/6,541)	**1.38 (1.15–1.65)**	<0.001/75.4	**1.48 (1.13–1.95)**	0.051/33.4	**1.41 (1.18–1.68)**	<0.001/76.6	**1.35 (1.05–1.73)**	0.090/28.3	**1.33 (1.15–1.53)**	<0.001/74.7
NDC	12 (3,133/14715)	1.18 (0.92–1.52)	<0.001/76.1	1.29 (0.81–2.08)	0.002/67.2	1.24 (0.94–1.63)	<0.001/82.5	1.05 (0.75–1.49)	0.033/52.3	1.18 (0.93–1.50)	<0.001/85.5
Matching											
Age and sex	41 (10103/20555)	**1.34 (1.15–1.56)**	<0.001/77.2	**1.30 (1.02–1.67)**	0.007/42.1	**1.37 (1.17–1.60)**	<0.001/79.6	1.13 (0.93–1.38)	0.079/27.4	**1.28 (1.13–1.46)**	<0.001/79.2
**Sensitivity analysis**											
**HWE**											
Overall	36 (9,817/20000)	**1.26 (1.10–1.45)**	<0.001/72.0	**1.30 (1.03–1.64)**	0.007/43.9	**1.29 (1.12–1.48)**	<0.001/74.5	1.21 (0.99–1.48)	0.061/31.1	**1.23 (1.10–1.38)**	<0.001/73.8
Ethnicity											
Asian	17 (3,636/3,428)	**1.44 (1.07–1.93)**	<0.001/76.7	1.04 (0.56–1.91)	0.594/0.0	**1.42 (1.06–1.91)**	<0.001/76.7	0.79 (0.46–1.36)	0.941/0.0	**1.29 (1.01–1.64)**	<0.001/72.1
Caucasian	11 (2,689/11805)	1.03 (0.94–1.14)	0.472/0.0	1.28 (0.87–1.87)	<0.001/70.1	1.11 (0.94–1.32)	0.022/51.9	1.23 (0.88–1.72)	0.002/64.8	1.13 (0.96–1.34)	<0.001/71.8
Type of control											
HC	27 (6,962/5,623)	**1.40 (1.16–1.68)**	<0.001/75.0	**1.50 (1.13–1.99)**	0.098/29.8	**1.43 (1.19–1.72)**	<0.001/76.3	**1.36 (1.06–1.75)**	0.178/22.0	**1.33 (1.14–1.53)**	<0.001/74.0
NDC	9 (2,855/14377)	0.97 (0.86–1.09)	0.306/15.4	0.99 (0.75–1.31)	0.242/24.4	0.97 (0.84–1.13)	0.126/36.5	1.01 (0.83–1.23)	0.389/5.0	0.99 (0.86–1.14)	0.049/48.6
Matching											
Age and sex	33 (9,198/19299)	**1.28 (1.10–1.49)**	<0.001/73.9	1.18 (0.96–1.45)	0.175/20.8	**1.29 (1.11–1.50)**	<0.001/75.3	1.11 (0.97–1.28)	0.517/0.0	**1.22 (1.08–1.37)**	<0.001/72.6
**Quality score >7**											
Overall	18 (5,533/15633)	1.09 (0.98–1.23)	0.134/27.6	1.01 (0.83–1.23)	0.416/3.1	1.20 (0.98–1.23)	0.085/33.2	1.02 (0.86–1.20)	0.500/0.0	1.07 (0.97–1.18)	0.074/34.7
Ethnicity											
Asian	8 (1,464/1,093)	**1.33 (1.07–1.66)**	0.538/0.0	0.84 (0.23–3.15)	0.882/0.0	**1.32 (1.06–1.65)**	0.617/0.0	0.81 (0.22–3.02)	0.856/0.0	**1.27 (1.03–1.56)**	0.736/0.0
Caucasian	6 (1926/10916)	0.99 (0.89–1.11)	0.726/0.0	0.90 (0.61–1.32)	0.087/50.8	0.99 (0.89–1.10)	0.433/0.0	0.91 (0.64–1.29)	0.119/45.5	0.98 (0.85–1.11)	0.174/35.0
Type of control											
HC	10 (2,710/1,382)	**1.28 (1.10–1.49)**	0.477/0.0	1.24 (0.76–2.02)	0.500/0.0	**1.28 (1.10–1.49)**	0.598/0.0	1.17 (0.72–1.89)	0.431/0.0	**1.22 (1.07–1.39)**	0.645/0.0
NDC	8 (2,823/14251)	0.98 (0.89–1.08)	0.472/0.0	0.95 (0.73–1.25)	0.271/21.6	0.97 (0.86–1.10)	0.247/22.9	0.99 (0.83–1.19)	0.415/0.1	0.98 (0.87–1.10)	0.124/38.3
Matching											
Age and sex	17 (5,282/15523)	1.11 (0.99–1.25)	0.122/29.6	1.04 (0.86–1.26)	0.431/1.5	1.12 (0.99–1.26)	0.086/33.8	1.04 (0.87–1.23)	0.495/0.0	1.09 (0.98–1.21)	0.083/34.1
**HWE and Quality score > 7**											
Overall	17 (5,449/15549)	1.10 (0.98–1.24)	0.102/31.9	1.01 (0.83–1.23)	0.416/3.1	1.10 (0.98–1.24)	0.063/37.1	1.02 (0.86–1.20)	0.500/0.0	1.08 (0.97–1.20)	0.054/38.5
Ethnicity											
Asian	8 (1,464/1,093)	**1.33 (1.07–1.66)**	0.538/0.0	0.84 (0.23–3.15)	0.882/0.0	**1.32 (1.06–1.65)**	0.617/0.0	0.81 (0.22–3.02)	0.856/0.0	**1.27 (1.03–1.56)**	0.736/0.0
Caucasian	5 (1842/10832)	0.99 (0.89–1.11)	0.591/0.0	0.90 (0.61–1.32)	0.087/50.8	0.98 (0.86–1.13)	0.305/17.2	0.91 (0.64–1.29)	0.119/45.5	0.97 (0.83–1.13)	0.105/47.8
Type of control											
HC	10 (2,710/1,382)	**1.28 (1.10–1.49)**	0.477/0.0	1.24 (0.76–2.02)	0.500/0.0	**1.28 (1.10–1.49)**	0.598/0.0	1.17 (0.72–1.89)	0.431/0.0	**1.22 (1.07–1.39)**	0.645/0.0
NDC	7 (2,739/14167)	0.97 (0.87–1.09)	0.365/8.3	0.95 (0.73–1.25)	0.271/21.6	0.97 (0.85–1.12)	0.172/33.5	0.99 (0.83–1.19)	0.415/0.1	0.98 (0.86–1.12)	0.079/46.9
Matching											
Age and sex	16 (5,198/15439)	1.12 (0.99–1.26)	0.090/34.0	1.04 (0.86–1.26)	0.431/1.5	1.12 (0.99–1.27)	0.063/37.9	1.04 (0.87–1.23)	0.495/0.0	1.10 (0.98–1.22)	0.060/38.2
Egger’s test											
*P*_E_		0.002		0.199		0.003		0.390		0.014	0.002

HC, health controls; NDC, Non-diabetic controls; HWE, Hardy–Weinberg equilibrium; *eNOS*, endothelial nitric oxide synthase. The bold values in table indicated that these results are statistically significant.

**FIGURE 4:**
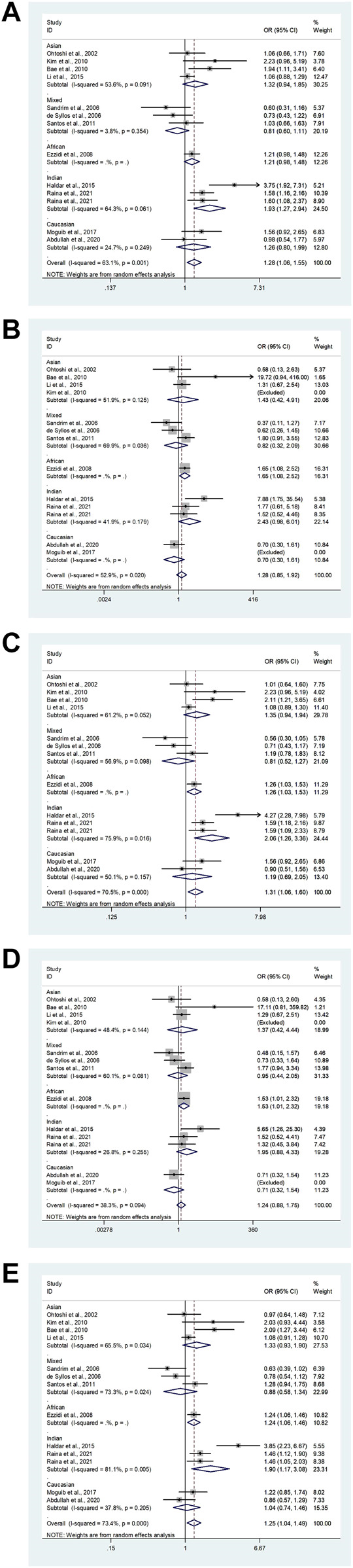
The forest plots of all selected studies on the association between eNOS t786C polymorphism and the risk of T2DM in different races [**(A)**: hybrid model; **(B)** homozygous model; **(C)** dominant model; **(D)** recessive model; **(E)** allele model].

Our study exposed an overall powerful association between *eNOS* T786C and T2DM susceptibility (TC vs. TT: OR = 1.28, 95%CI = 1.06–1.55; TC + CC vs. TT: OR = 1.31, 95%CI = 1.06–1.60; C vs. T: OR = 1.25, 95%CI = 1.04–1.49, Table 8; Figure 4). At the same time, subgroup studies revealed that Indians had a significantly increased risk of T2DM (TC vs. TT: OR = 1.93, 95% CI = 1.27–2.94; TC + CC vs. TT: OR = 2.06, 95%CI = 1.26–3.36; C vs. T: OR = 1.90, 95%CI = 1.17–3.08, Table 8; Figure 4). Moreover, no signification association was observed in the healthy population according to type of control (Table 8).

**TABLE 8:** Meta-analysis of the association of *eNOS* T786C polymorphism with risk of T2DM.

Variable	n (Cases/Controls)	TC vs. TT		CC vs. TT		TC + CC vs. TT		CC vs. (TT + TC)		C vs. T	
		Or (95%CI)	*P*_h_/*I* ^2^ (%)	Or (95%CI)	*P*_h_/*I* ^2^ (%)	Or (95%CI)	*P*_h_/*I* ^2^ (%)	Or (95%CI)	*P*_h_/*I* ^2^ (%)	Or (95%CI)	*P*_h_/*I* ^2^ (%)
Overall	13 (4,676/3,842)	1.28 (1.06–1.55)	0.001/63.1	1.28 (0.85–1.93)	0.020/52.9	**1.31 (1.06–1.60)**	<0.001/70.5	1.24 (0.88–1.75)	0.094/38.3	**1.25 (1.04–1.49)**	<0.001/73.4
Ethnicity											
Asian	4 (1,660/1974)	1.32 (0.94–1.85)	0.091/53.6	1.43 (0.42–4.91)	0.125/51.9	1.35 (0.94–1.94)	0.052/61.2	1.37 (0.42–4.44)	0.144/48.4	1.33 (0.93–1.90)	0.034/65.5
Indian	3 (943/615)	1.93 (1.27–2.94)	0.061/64.3	2.43 (0.98–6.01)	0.179/41.9	**2.06 (1.26–3.36)**	0.016/75.9	1.95 (0.88–4.33)	0.255/26.8	**1.90 (1.17–3.08)**	0.005/81.1
Type of control											
Healthy controls	11 (4,551/3,373)	1.22 (0.99–1.48)	0.002/64.0	1.22 (0.83–1.81)	0.033/50.5	1.23 (0.99–1.52)	<0.001/71.5	1.21 (0.88–1.67)	0.146/32.8	1.18 (0.98–1.41)	<0.001/74.0
Sensitivity analysis											
HWE											
Overall	12 (4,476/3,742)	1.27 (1.03–1.55)	0.001/65.3	1.28 (0.85–1.93)	0.020/52.9	**1.29 (1.04–1.60)**	<0.001/72.5	1.24 (0.88–1.75)	0.094/38.3	**1.25 (1.03–1.51)**	<0.001/75.6
Ethnicity											
Asian	4 (1,660/1974)	1.32 (0.94–1.85)	0.091/53.6	1.43 (0.42–4.91)	0.125/51.9	1.35 (0.94–1.94)	0.052/61.2	1.37 (0.42–4.44)	0.144/48.4	1.33 (0.93–1.90)	0.034/65.5
Indian	3 (943/615)	1.93 (1.27–2.94)	0.061/64.3	2.43 (0.98–6.01)	0.179/41.9	**2.06 (1.26–3.36)**	0.016/75.9	1.95 (0.88–4.33)	0.255/26.8	**1.90 (1.17–3.08)**	0.005/81.1
Type of control											
Healthy controls	10 (4,351/3,273)	1.19 (0.97–1.47)	0.002/66.4	1.22 (0.83–1.81)	0.033/50.5	1.21 (0.96–1.51)	<0.001/73.7	1.21 (0.88–1.67)	0.146/32.8	1.18 (0.97–1.43)	<0.001/76.6
Quality score >7											
Overall	5 (1,649/1,014)	1.70 (1.23–2.35)	0.036/61.0	**2.32 (1.25–4.33)**	0.220/30.2	**1.82 (1.31–2.55)**	0.019/66.0	**1.97 (1.16–3.37)**	0.314/15.8	**1.74 (1.28–2.36)**	0.008/70.7
Ethnicity											
Indian	3 (943/615)	1.93 (1.27–2.94)	0.061/64.3	2.42 (0.98–6.01)	0.179/41.9	**2.06 (1.26–3.36)**	0.016/75.9	1.95 (0.88–4.33)	0.255/26.8	**1.90 (1.17–3.08)**	0.005/81.1
Type of control											
Healthy controls	4 (1,560/715)	1.67 (1.13–2.47)	0.020/69.4	**2.07 (1.20–3.58)**	0.305/17.2	**1.79 (1.20–2.66)**	0.011/73.0	**1.82 (1.14–2.89)**	0.439/0.0	**1.69 (1.19–2.40)**	0.006/75.7
HWE and Quality score >7											
Overall	5 (1,649/1,014)	1.70 (1.23–2.35)	0.036/61.0	**2.32 (1.25–4.33)**	0.220/30.2	**1.82 (1.31–2.55)**	0.019/66.0	**1.97 (1.16–3.37)**	0.314/15.8	**1.74 (1.28–2.36)**	0.008/70.7
Ethnicity											
Indian	3 (943/615)	1.93 (1.27–2.94)	0.061/64.3	2.42 (0.98–6.01)	0.179/41.9	**2.06 (1.26–3.36)**	0.016/75.9	1.95 (0.88–4.33)	0.255/26.8	**1.90 (1.17–3.08)**	0.005/81.1
Type of control											
Healthy controls	4 (1,560/715)	1.67 (1.13–2.47)	0.020/69.4	**2.07 (1.20–3.58)**	0.305/17.2	**1.79 (1.20–2.66)**	0.011/73.0	**1.82 (1.14–2.89)**	0.439/0.0	**1.69 (1.19–2.40)**	0.006/75.7
Egger’s test											
***P***_***E***_		0.420		0.941		0.498		0.905		0.517	

HWE, Hardy–Weinberg equilibrium; *eNOS*, endothelial nitric oxide synthase. The bold values in table indicated that these results are statistically significant.

### Heterogeneity and Sensitivity Analyses

Several possible causes of variation were discovered, including ethnicity, gender, sample size, age, quality score, type of controls and HWE. Therefore, a meta-regression analysis was used to identify causes of heterogeneity. For the *eNOS* G894T, no covariate was found as a possible cause of between-study variation. A meta-regression analysis revealed that HWE (ab vs. aa: *p* = 0.045) was the source of heterogeneity between the *eNOS* 4b/a polymorphism and the risk of T2DM. At the same time, the quality score (TC vs. TT: *p* = 0.045; TC + CC vs. TT: *p* = 0.042; C vs. T: *p* = 0.041) and HWE (CC vs. TT: *p* = 0.029; CC vs. TC + TT: *p* = 0.041) were the sources of heterogeneity between the *eNOS* T786C polymorphism and the risk of T2DM.

Three methods were employed for sensitivity analyses in this meta-analysis. Firstly, results did not alter when a single study was removed each time. Second, when HWD studies were omitted, Asians were found to have a significantly lower risk of *eNOS* 4b/a polymorphism and T2DM in the overall analysis (Table 6). For the *eNOS* G894T polymorphism, significantly increased T2DM risk was only observed in Asians and healthy population when we retained high-quality and HWE studies in the control group (Table 7). For the *eNOS* T786C polymorphism, a significant association was also discovered in the healthy population when we only included high-quality and HWE studies in the control group (Table 8).

### Publication Bias

**FIGURE 5:**
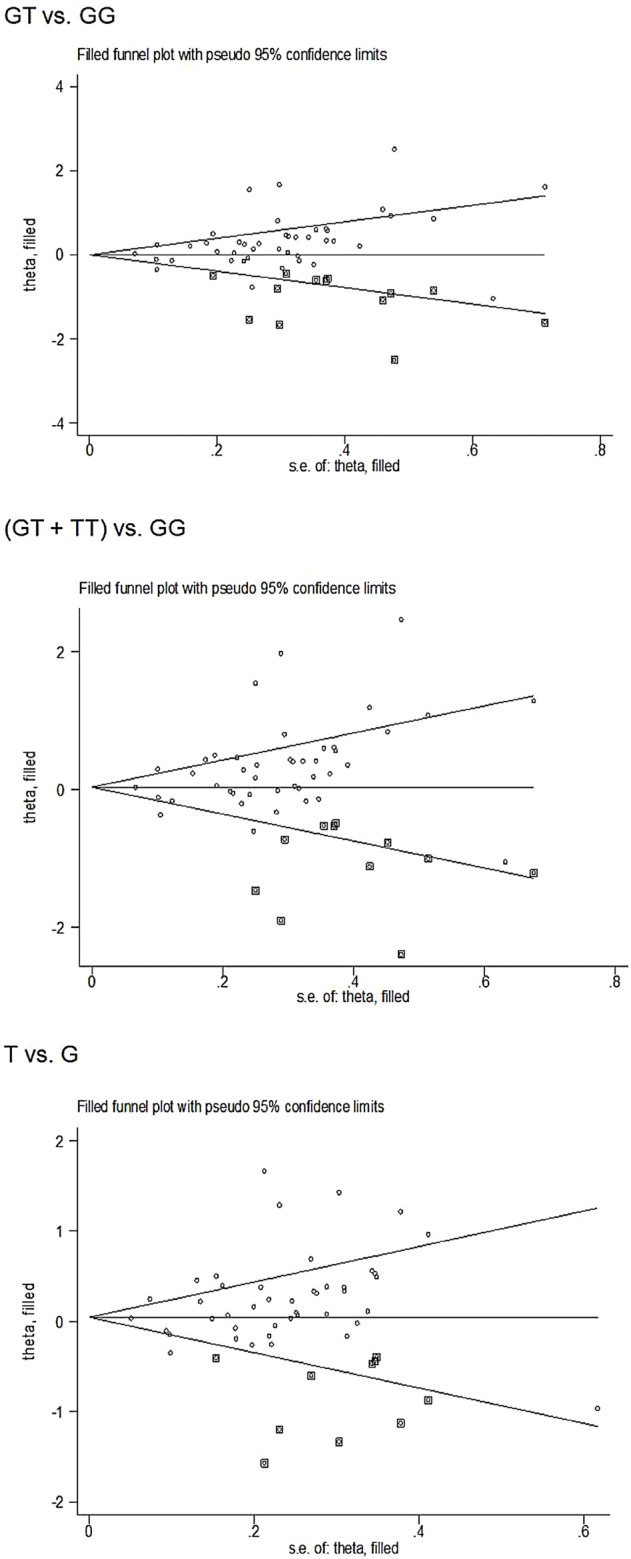
Begg’s funnel plot to assess publication bias.

Begg’s funnel plot and Egger’s test revealed only publication bias between the *eNOS* G894T polymorphism and T2DM risk (GT vs. GG: *p* = 0.002; GT + TT vs. GG: *p* = 0.003; T against G: *p* = 0.014, Table 7). Then, publication bias was adjusted using the nonparametric “trim and fill” method. And we need to add 13, 11, and 10 articles in the future for GT vs. GG, GT + TT vs. GG, and T vs. G models, respectively (Figure 5). In the overall analysis, the findings for GT vs. GG, GT + TT vs. GG, and T vs. G models did not change (data not shown), demonstrating that more research cannot alter the merger outcomes.

### TSA Results

**FIGURE 6:**
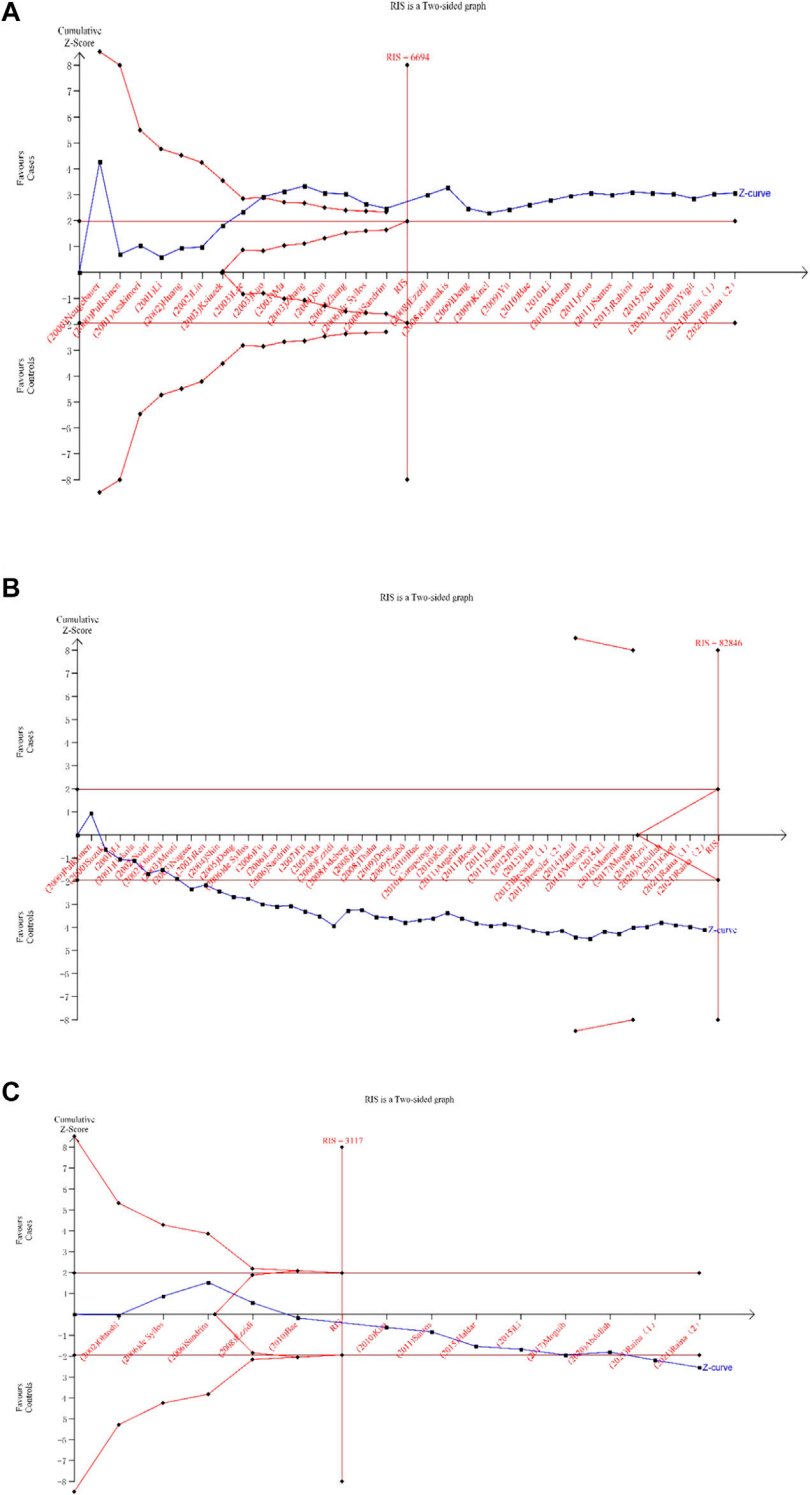
Trial sequential analysis for the eNOS polymorphisms under dominant gene model [**(A)**: (bb + ab) vs. aa; **(B)** (TT + GT) vs. GG; **(C)** (CC + TC) vsTT)].

The TSA of the dominant model for the *eNOS* 4b/a and T786C polymorphisms revealed that the cumulative z-curve passed both the RIS line and the TSA threshold, indicating that no more evidence was required to confirm the conclusion. However, multiple comparisons and other confounding factors, we believe, can still increase the occurrence of false positive errors, so credibility analysis is still required for the *eNOS* 4b/a and T786C polymorphisms. The cumulative Z-curve of the dominant model for the *eNOS* G894T polymorphism did not surpass the TSA threshold, and the total number of cases and controls was smaller than the RIS, according to the TSA. Therefore, more trials were still required to confirm the association between *eNOS* G894T polymorphism and T2DM risk. Figure 6 displays the above results.

### Credibility of the Identified Genetic Associations

The credibility of this meta-analysis was assessed using the FPRP, BFDP, and Venice criteria. Associations meeting the following criteria were regarded to be of high credibility [29]: 1) statistically significant associations were observed in at least two of the genetic models; 2) FPRP <0.2 and BFDP <0.8; 3) *I* ^2^ < 50%; and 4) statistical power >80%. All other major findings were viewed as “less credible results”. All statistically significant associations were deemed “less credible” in this study.

## Discussion

T2DM is a polygenic genetic disease, which is also greatly influenced by environmental factors. And it is the outcome of the combined action of numerous genes and environmental factors. Several studies have shown that diabetes is the most important risk factor for mortality and disability caused by cardiovascular and cerebrovascular illnesses, according to several research. However, the molecular mechanism of the genetics of T2DM has not been elucidated. Much significant evidence indicates that the *eNOS* polymorphisms have been considered as potential genetic factors for T2DM. Numerous *eNOS* polymorphisms have been reported, and their relationship with various disorders has been studied, including coronary artery disease, myocardial infarction, coronary spasm, hypertension, end-stage renal disease (ESRD), and T2DM. Previous research has focused on three *eNOS* polymorphisms: the intronic 427-bp repeat (4b/a) in the promoter region; the *G894T* (Glu298Asp) missense mutation in exon seven and the T786C single nucleotide polymorphism. T786C inhibits *eNOS* transcription, G894T inhibits *eNOS* activity, and 4b/a inhibits plasma NO concentrations, which may be a reflection of *eNOS* activity. Many researchers have sought to investigate the potential relationship between *eNOS* polymorphisms and T2DM risk. Regrettably, no credible evidence is available, which might be attributable to a variety of factors such as small sample numbers, ethnic and geographical disparities. As a result, meta-analysis is an effective method to conquer these flaws.

Overall, the *eNOS* 4b/a was connected with a substantially lower the risk of T2DM in Asians; the *eNOS* G894T was connected with a significantly higher risk of T2DM in Asians, however, it had no significant effect on the risk of T2DM in Caucasians. the *eNOS* T786C was connected with a significantly higher risk of T2DM in Indians. However, after omitting low-quality and HWD studies, we observed that *eNOS* 4b/a polymorphism substantially lowered T2DM risk in the entire population while *eNOS* T786C polymorphism considerably raised T2DM risk in the whole population. However, after omitting low-quality and HWD studies, we observed that *eNOS* 4b/a polymorphism substantially lowered T2DM risk in the entire population while *eNOS* T786C polymorphism considerably raised T2DM risk in the whole population. The current study used many subgroups and distinct genetic models, which resulted in multiple comparisons, so the pooled *p* value must be corrected. FPRP has been described as a proper method for assessing the likelihood of significant outcomes in molecular epidemiology investigations using multiple hypothesis testing. Furthermore, Wakefield suggested a more accurate Bayesian metric of false detection in genetic epidemiology investigations in 2007. Many factors may lead to errors and biases, such as genotyping errors and phenotypic misclassification, of which statistical power was a significant factor. A substantial amount of evidence (statistical power>80%) can achieve a higher degree of statistical significance or reduce the false-discovery rate. As a result, in this study, we used the FPRP test, BFDP test and the Venice criteria to evaluate false discovery. All the statistically significant connections were less-credible in the current meta-analysis after assessing credibility. Our meta-analysis has also revealed heterogeneity. According to the results of the meta-regression study, the quality score and HWE were the sources of heterogeneity. Furthermore, bias and mistakes were widespread in several HWD studies with low quality and small sample size, making the conclusion of these original studies untrustworthy, particularly in molecular epidemiology studies. And small sample studies with positive results may be easier to accept since they are likely to produce false-positive results because their research is less rigorous and frequently of poor quality. The asymmetry of the funnel plot was created by a study of low-quality small samples. Therefore, we added high-quality and HWE to evaluate sensitivity analyses in control studies.

We hypothesized that the single and combined effects of the *eNOS* 4b/a, G894T, and T786C polymorphisms were linked with T2DM risk in all races based on the biochemical features outlined for these genes. Nevertheless, when we applied the FPRP, BFDP test, and Venice criteria to assess the credibility of this meta-analysis, all statistically significant relationships were declared “less credible” (greater heterogeneity, FPRP >0.2, BRDP >0.8, and lower statistic power). Therefore, these results indicated that much larger sample size was needed to study the potential gene-gene interactions.

A total of three previously published meta-analyses investigated the relationship between the *eNOS* 4b/a, G894T, and T786C polymorphisms and the risk of T2DM. There was a clear mismatch in the categorization of ethnic groupings between the previous related meta-analyses and the current meta-analysis. Furthermore, the sample size in this study was substantially greater. A total of 66 articles were included in this study, of which 36 articles reported the *eNOS* 4b/a (8,553 cases and 6,613 controls), 44 articles investigated the *eNOS* G894T (10,722 cases and 21,256 controls), and 13 articles reported the *eNOS* T786C (4,676 cases and 3,842 controls). Five genetic models were compared separately in this study. However, Dong et al., Zhang et al. and Jia et al. applied four genetic models. In addition, when we used the FPRP, BFDP test, and Venice criteria to assess the credibility of the previous meta-analyses, all statistically significant relationships were deemed “less credible.” As a result, their findings may be unreliable.

Compared with the previous meta-analysis, the new meta-analysis had several advantages: 1) credibility was investigated using FPRP, BFDP test and Venice criteria; 2) the quality of the eligible research was evaluated; 3) The sample size was larger and the data collected was more detailed than the previous meta-analyses; 4) more subgroup analyses were performed according to the type of control, matching and quality score; 5) TSA was carried out to decrease random mistakes. However, there are still some potential limitations in the current meta-analysis. First, the current meta-analysis included only published research, although positive outcomes are known to be published more frequently than negative ones. Second, T2DM is a complex multi-genetic disorder, and the link between an individual SNP and T2DM risk is relatively weak. However, we did not retrieve the corresponding data on the combined impacts of gene-gene and gene-environment. Third, the relationship between the *eNOS* polymorphisms and the risk of T2DM complications has not been investigated. Therefore, the current meta-analysis has a large sample size and a sufficiently large subgroup to help confirm our findings.

To summarize, our study reveals that all substantial relationships between the *eNOS* 4b/a, G894T, and T786C polymorphisms and T2DM risk are most likely due to false-positive results rather than real connections or biological variables. larger-scale epidemiological studies on this topic should be conducted in the future to confirm or disprove our findings.

## Data Availability

The original contributions presented in the study are included in the article/Supplementary Material, further inquiries can be directed to the corresponding authors.

## Abbreviations

AIS, accruing information size; BFDP, Bayesian false discovery probability; CIs, confidence intervals; CNKI, China National Knowledge Infrastructure; eNOS, endothelial nitric oxide synthase; FPRP, false-positive report probability; HWD, Hardy–Weinberg Disequilibrium; HWE, Hardy–Weinberg equilibrium; ISI; International Statistical Institute; NOS, Newcastle-Ottawa Scale; NO, Nitric oxide; ORs, odds ratios; RIS, required information size; RRR, relative risk reduction; T2DM, type 2 diabetes mellitus; TSA, Trial sequential analysis.
